# The effects of a graduated aerobic exercise programme on cardiovascular disease risk factors in the NHS workplace: a randomised controlled trial

**DOI:** 10.1186/1745-6673-3-7

**Published:** 2008-02-28

**Authors:** Jennifer A Hewitt, Gregory P Whyte, Michelle Moreton, Ken A van Someren, Tanya S Levine

**Affiliations:** 1 Kingston University, Kingston Upon Thames, UK; 2 St George's, University Of London, Tooting, UK; 3 North West London Hospitals NHS Trust, Harrow, UK; 4 Liverpool John Moores University, Liverpool, UK; 5 English Institute Of Sport, Twickenham, UK

## Abstract

**Background:**

Sufficient levels of physical activity provide cardio-protective benefit. However within developed society sedentary work and inflexible working hours promotes physical inactivity. Consequently to ensure a healthy workforce there is a requirement for exercise strategies adaptable to occupational time constraint. This study examined the effect of a 12 week aerobic exercise training intervention programme implemented during working hours on the cardiovascular profile of a sedentary hospital workforce.

**Methods:**

Twenty healthy, sedentary full-time staff members of the North West London Hospital Trust cytology unit were randomly assigned to an exercise (n = 12; mean ± SD age 41 ± 8 years, body mass 69 ± 12 kg) or control (n = 8; mean ± SD age 42 ± 8 years, body mass 69 ± 12 kg) group. The exercise group was prescribed a progressive aerobic exercise-training programme to be performed 4 times a week for 8 weeks (initial intensity 65% peak oxygen consumption (VO_2 peak_)) and to be conducted without further advice for another 4 weeks. The control was instructed to maintain their current physical activity level. Oxygen economy at 2 minutes (2minVO_2_), 4 minutes (4minVO_2_), VO_2 peak_, systolic blood pressure (SBP), diastolic blood pressure (DBP), BMI, C-reactive protein (CRP), fasting glucose (GLU) and total cholesterol (TC) were determined in both groups pre-intervention and at 4 week intervals. Both groups completed a weekly Leisure Time Questionnaire to quantify additional exercise load.

**Results:**

The exercise group demonstrated an increase from baseline for VO_2 peak _at week 4 (5.8 ± 6.3 %) and 8 (5.0 ± 8.7 %) (P < 0.05). 2minVO_2 _was reduced from baseline at week 4 (-10.2 ± 10.3 %), 8 (-16.8 ± 10.6 %) and 12 (-15.1 ± 8.7 %), and 4minVO_2 _at week 8 (-10.7 ± 7.9 %) and 12 (-6.8 ± 9.2) (P < 0.05). There was also a reduction from baseline in CRP at week 4 (-0.4 ± 0.6 mg·L^-1^) and 8 (-0.9 ± 0.8 mg·L^-1^) (P < 0.05). The control group showed no such improvements.

**Conclusion:**

This is the first objectively monitored RCT to show that moderate exercise can be successfully incorporated into working hours, to significantly improve physical capacity and cardiovascular health.

## Background

It is widely accepted that cardiovascular disease (CVD) is the leading cause of death in developed countries [1]. Over the past decade it has become recognised that physical activity is an independent factor in the determination of over all CVD risk through the prevention of atherosclerosis and reduction of thrombotic risk [2,3]. Evidence supports an inverse association between physical fitness and various CVD risk factors, including glucose tolerance [4], cholesterol [5], blood pressure [6], resting pulse rate [7] and obesity [8], and markers of systemic inflammation including C-reactive protein (CRP) [9], and TNFα [10]. It is suggested that such effects occur through a reduction in lipoprotein oxidation [11], improved endothelial function via the increased production of nitric oxide and prostacyclin [12], decreased atherogenic activity of blood mononuclear cells effecting the production of cytokines [13], and a reduced accumulation of collagen in the arterial wall [14]. Therefore guidelines recommend that individuals accrue 30 minutes of moderate physical activity on at least 5 days of the week [15,16].

Despite the positive impact of physical fitness on CVD, developed societies have become more sedentary in both occupation and leisure time. A recent observational study of 2595 civil servants in Northern Ireland reported that almost two thirds failed to engage in regular, moderate physical activity, with females twice as likely to abstain from exercise than men [17]. In England it has been reported that a total of 24.2% of men and 19.8% of women meet the activity recommendations; a total that dropped to 17.6% and 13.0% when domestic activity was excluded [18]. Since most adults will spend more than half their waking hours within the workplace, worksite health promotion programs that influence employee behaviour by promoting physical activity could prove fundamental in addressing the growing problem of sedentary habit and cardiovascular risk.

A number of randomised-controlled trials assessing the benefit of workplace exercise interventions on health-related outcome measures (body composition, blood pressure, lipid profile, inflammatory markers) have been reported [19-21]. However, the conclusions from these trials have been based upon the subjective self-report of physical activity, without individualised prescription or monitoring of the exercise programme, and objective assessment. Therefore the relationship between improved physical capacity and health from workplace exercise remains inconclusive [21]. In view of this there is a necessity for further studies of strong methodological quality to examine corporate exercise strategies adaptable to occupational time constraints.

The aim of this pilot study was to investigate the efficacy of a structured, monitored 12-week aerobic exercise training intervention programme on modifying the cardiovascular risk profile of a sedentary National Health Service (NHS) workforce, and to evaluate whether it could be implemented during working hours.

## Methods

### Setting

The trial was conducted at the Olympic Medical Institute (OMI), Northwick Park and North West London Hospitals (NWLH) NHS Trust (Northwick Park site). The North West London Research Ethics Committee, NWLH NHS Trust approved the trial (REC 05/Q0405/122). All participants provided written informed consent before entering the study.

### Study participants

Participants were full-time male and female personnel from the NWLH Trust cytology laboratory. Who as specialist medical and non-medical cytology staff, spend multiple hours per day seated for the microscopic assessment of cervical cytology slides. All subjects were defined "sedentary" from self-reported physical activity levels of less than 2 hours organised physical activity per week. Eligible participants were not admitted if they had known cardiac disease, uncontrolled hypertension, thyroid disease, diabetes, mental illness, infection, immune or endocrine abnormality or contraindications to exercise on the basis of an exercise stress test. All participants were required to complete a medical screening questionnaire (PAR-Q) before entering the study.

20 participants were recruited and randomly assigned to an exercise (n = 12) or control (n = 8) group using a random numbers table. Group assignment was revealed following baseline testing.

### Experimental design

**Figure 1:**
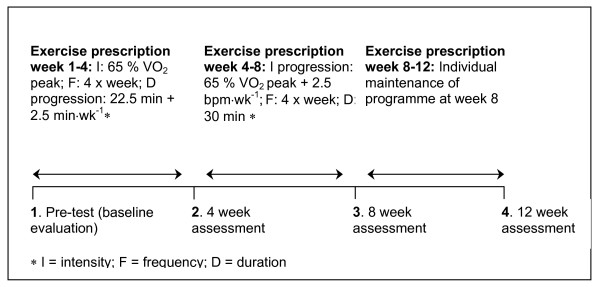
Schematic experimental time-line of the aerobic exercise training intervention programme.

Physiological tests included blood pressure, body composition, peak oxygen uptake and blood screening, and were performed at pre-intervention and at 4 weekly intervals for a total of 12 weeks. After baseline assessment and at each 4 week reassessment, control subjects were instructed to maintain their current physical activity level, while the exercise group were provided with an individualised progressive exercise prescription of brisk walking or light jogging to be performed 4 times a week for the following 4 weeks (Figure 1.). At 8 weeks no further progression of the exercise training programme was provided, and participants were instructed to maintain the exercise as of week 8 for the final 4 weeks. This was to evaluate if there was any further physiological benefit, or if exercise adherence was affected in the absence of any additional training stimulus. Participants conducted all exercise sessions during their lunch, morning or afternoon breaks, to avoid disturbance to the normal laboratory working routine. Heart rate monitors (F4, Polar electro-oy, Kempele, Finland) were provided to monitor accurately the intensity of the exercise prescribed, and the average heart rate and exercise duration of each session was recorded in an exercise diary. The exercise intensity was initially set to correspond with 65 % of peak oxygen consumption (VO_2 peak_). Participants were instructed on an appropriate warm-up and cool-down procedure, and provided with a supervised exercise session during the initial week of each 4 week period. Progress was checked through personal contact on a weekly basis. At each exercise testing session all participants were provided with an evaluation of their results.

Both groups were provided with the Godin Leisure Time Questionnaire [22] to record in arbitrary units any additional physical activity or exercise that was above the prescribed programme. On entering the study participants were asked to complete a typical retrospective week of the same questionnaire. This was to ensure that all individuals from both groups participated in similar amounts of physical activity or exercise at baseline. The control group was offered the intervention at the end of the trial.

### Outcomes

The primary outcomes were changes in peak oxygen consumption (VO_2 peak_), submaximal oxygen consumption at 2 minutes (2minVO_2_) and 4 minutes (4minVO_2_), and biological markers of inflammation (C-reactive protein, IL-6 and TNFα) between baseline and post intervention. Secondary outcomes were changes in time to exhaustion, resting heart rate, systolic and diastolic blood pressure. Secondary biological markers were fasting glucose and total cholesterol. Secondary physical outcomes were changes in body weight and body mass index (BMI). All outcome measures were taken after a 24 hour period of no exercise.

### Biological outcomes

Fasting blood samples were collected in the morning, before any of the physiological tests. Whole blood samples were analysed for total cholesterol and glucose using an Abbott 8200 analyser (Abbott, Chicago, IL, USA). Cholesterol and glucose levels were measured using the cholesterol oxidase and hexokinase method respectively. Serum samples were used for CRP, TNFα, and IL-6. These were separated by low-speed centrifugation, and stored for later analysis at -70°C. The assays were performed using a semi-automated solid-phase, enzyme-labelled, chemiluminescent sequential immunometric assay (Euro/DPC, Gwynedd, UK), and measured using an IMMULITE 1000 analyser (Immulite, Gwynedd, UK). The lowest detection levels for IL-6, TNFα and CRP were 2 pg/mL, 1.7 pg/mL and 0.1 mg/L respectively. For the purpose of data analysis all values below the detection limit were coded as 1.9 pg/mL, 1.6 pg/mL and 0.05 mg/L respectively.

### Blood pressure

Subjects remained in the supine position for 10 minutes. Blood pressure was measured manually, and recorded to the nearest 2 mm Hg. Each measurement was repeated three times then averaged.

### Physical characteristics

Body composition was assessed indirectly through changes in body weight and body mass index. Body weight was assessed using an electronic scale (Seca, Vogel Halke, Germany). Standing height was determined without shoes. Body mass index was calculated as body mass (Kg) divided by height squared (m^2^).

### Cardiopulmonary outcomes

Cardiopulmonary outcomes were evaluated using a progressive walking test (modified Bruce protocol) to volitional fatigue on a motorised treadmill. Speed (2.5, 3, 3.5 or 4 m·p^-1· ^h^-1^) was predetermined by the participant's previous exercise history, and remained constant for the duration of the test, and for each subsequent test. The gradient was set at 2 % and increased by 1 % each minute. Heart rate data were recorded at 1-minute intervals. On the initial test this was used with VO_2 _data to determine the heart rate training intensity (65 % VO_2 peak_) of the exercise-training programme. This procedure was repeated at 4 and 8 weeks to ensure correct continuation of the heart rate training prescription. Participants were provided with standardized encouragement throughout the test.

Criteria for peak oxygen consumption included any two of the following: a peak or plateau for more than 1 minute in oxygen consumption; a respiratory exchange ratio ≥ 1.15; volitional exhaustion; and rating of perceived exertion greater than 19 (Borg, 1980). Exercise was terminated if participants developed severe dyspnea, dizziness, or chest pain, or had an abnormal heart rate response.

Expired gases were analysed every 5 seconds using an automated online gas analyser (Oxycon, Jaeger, Hoechberg, Germany). The system was calibrated for volume and gas concentrations before the start of each test. Peak oxygen consumption and oxygen consumption at 2 and 4-minute intervals were determined by taking the mean of twelve consecutive 5-second values at the end of each respective stage. Participants were asked to follow the same diet for the 24 hour period preceding each testing session.

### Statistical analysis

Baseline characteristics between groups were compared using independent-samples t tests. Cardiopulmonary outcomes were normalized to baseline, and expressed as percentage change. Due to skewed distribution CRP data was log transformed. Repeated measures ANOVA were used to determine differences in outcomes between groups. Post hoc analysis was made within groups between each time-point. Where significant interaction effects were found, post hoc analysis was made at each time point between groups. SPSS version 14.0 (SPSS Inc, Chicago, IL, USA) was used for all statistical analyses. A P value < 0.05 was considered to be statistically significant. The results are reported as mean ± SD values.

## Results

### Baseline characteristics

Table 1 presents the baseline characteristics of the exercise (n = 12) and the control (n = 8) groups. There were no significant differences between groups for baseline characteristics.

**Table 1:** Baseline characteristics of exercise and control groups

Characteristic	Exercise Group (n = 12)	Control Group (n = 8)	≠ *P *value
Age (yrs)	41 ± 8	42 ± 8	0.460
Weight (kg)	68.5 ± 12.1	66.4 ± 13.2	0.659
BMI	25.9 ± 4.4	26 ± 4.1	0.777
Diastolic BP (mm Hg)	73 ± 10	69 ± 9	0.569
Systolic BP (mm Hg)	118 ± 12	106 ± 10	0.082
Resting heart rate (bpm)	66 ± 9	67 ± 11	0.821
Peak heart rate (bpm)	179 ± 14	182 ± 11	0.893
Time to exhaustion (min)	11.1 ± 3.5	10.7 ± 2.1	0.796
VO_2 peak _(L·min^-1^)	2.31 ± 0.65	2.00 ± 0.58	0.244
VO_2 peak _(mL·kg·min^-1^)	33.7 ± 8.8	35.5 ± 8.6	0.593
2 min oxygen consumption (L·min^-1^)	1.6 ± 0.49	1.3 ± 0.35	0.099
2 min oxygen consumption (mL·kg·min^-1^)	23.1 ± 5.2	20.4 ± 4.6	0.202
4 min oxygen consumption (L·min^-1^)	1.6 ± 0.36	1.4 ± 0.40	0.524
4 min oxygen consumption (mL·kg·min^-1^)	23.9 ± 4.5	23.0 ± 4.5	0.200
Past exercise (Godin arbitary units)	6.5 ± 4	7.5 ± 5.5	0.893
Total Cholesterol (mmol/L)	5.13 ± 1.0	4.97 ± 0.9	0.728
Glucose (mmol/L)	5.04 ± 0.50	5.11 ± 0.52	0.763
C-reactive protein (mg/L)	3.05 ± 4.37	3.16 ± 4.73	0.689
Interleukin-6 (pg/mL)	3.21 ± 0.91	3.26 ± 1.08	0.479
TNF-α (pg/L)	12.07 ± 3.27	9.84 ± 2.59	0.082

*Data are presented as mean (SD).

### Adherence to the exercise training intervention

The exercise group completed 81 ± 14 % (13 ± 2), 84 ± 12 % (13 ± 2) and 70 ± 13 % (11 ± 2) of the 16 prescribed exercise sessions between week 1 and week 4, week 4 and week 8, and week 8 and week 12 respectively. Non-protocol related exercise was not significantly different between groups at any time point during the study (week 4 P = 0.893; week 8 P = 0.952; week 12 P = 0.941).

### Changes in cardiopulmonary function

Table 2 and 3 present the cardiopulmonary outcomes. There was no significant time effect (F = 1.752; P = 0.167) in VO_2 peak _(L·min^-1^), but there was a significant interaction effect (F = 8.351; P = 0.000) and a treatment effect (F = 25.147; P = 0.000) between exercise and control groups. Post hoc analysis revealed that there were significant differences between exercise and control groups at all time points tested (P = 0.001; P = 0.001; P = 0.000). Furthermore, in the exercise group VO_2 peak _(L·min^-1^) significantly increased between week 0 and week 4 (P = 0.012), while in the control group it significantly decreased between week 0 and week 4 (P = 0.026), week 0 and week 8 (P = 0.004) and week 0 and 12 (P = 0.001) respectively. However, while there were no significant differences in peak heart rate (HRP) from baseline to any of the time points tested in the exercise group, HRP in the control group was significantly lower at all time points (P = 0.015; P = 0.032; P = 0.001). There was no significant time effect in time to exhaustion (TE) (F = 1.283; P = 0.334), but there were significant interaction and treatment effects between the exercise and the control conditions (F = 4.239; P = 0.006; F = 12.289; P = 0.002). Post hoc analysis between groups revealed significant differences at weeks 4 (P = 0.003), 8 (P = 0.002) and 12 (P = 0.036) respectively. Furthermore in the exercise group TE significantly increased from week 0 – week 4 (P = 0.005), week 0 – week 8 (P = 0.002) and week 0 – week 12 (P = 0.025), but no significant changes occurred in the control group at any time point.

**Table 2:** Effects of the exercise-training programme on physiological outcomes from baseline – exercise group (n = 12); control group (n = 8)

	**% Δ Week 1 – 4**			**% Δ Week 1 – 8**			**% Δ Week 1 – 12**		
**Variable**	**Exercise (mean ± SD)**	**Control (mean ± SD)**	**Difference between groups**	**Exercise (mean ± SD)**	**Control (mean ± SD)**	**Difference between groups**	**Exercise (mean ± SD)**	**Control (mean ± SD)**	**Difference between groups**

**Peak oxygen consumption (mL·min)**	5.8 ± 6.3 P = 0.012 (122 ± 142)	-3.7 ± 4.4 P = 0.026 (-69 ± 80)	P≠ = 0.001	5.0 ± 8.7 P = 0.032 (137 ± 190)	-6.0 ± 5.8 P = 0.004 (-107 ± 93)	P≠ = 0.001	2.1 ± 8.5 P = 0.105 (103 ± 208)	-8.2 ± 5.4 P = 0.001 (-153 ± 105)	P≠ = 0.000
**Peak oxygen consumption (mL·kg·min^-1^)**	6.0 ± 7.2 P = 0.029 (1.6 ± 2.2)	-4.8 ± 3.3 P = 0.005 (-1.4 ± 0.9)	P≠ = 0.000	5.3 ± 10.0 P = 0.063 (1.8 ± 3.2)	-5.8 ± 5.2 P = 0.002 (-1.7 ± 1.5)	P≠ = 0.003	1.6 ± 9.9 P = 0.200 (1.3 ± 3.8)	-8.9 ± 5.0 P = 0.350 (-2.8 ± 1.9)	P≠ = 0.001
**Time to exhaustion (min)**	12.5 ± 12.5 P = 0.005 (1.1 ± 1.7)	-6.9 ± 12.2 P = 0.157 (-0.6 ± 1.2)	P≠ = 0.003	16.7 ± 14.7 P = 0.002 (1.5 ± 1.6)	-7.9 ± 14.0 P = 0.158 (-0.9 ± 1.7)	P≠ = 0.002	16.5 ± 22.0 P = 0.025 (1.4 ± 3.0)	-3.6 ± 14.6 P = 0.506 (-0.48 ± 1.42)	P≠ = 0.036
**Peak heart rate (bpm)**	0.1 ± 2.5 P = 0.872 (0 ± 4)	-1.7 ± 1.5 P = 0.015 (3 ± 3)	P≠ = 0.072	-1.07 ± 3.79 P = 0.291 (-2 ± 7)	-2.43 ± 2.56 P = 0.032 (-5 ± 5)	P≠ = 0.405	0.01 ± 3.34 P = 0.931 (0 ± 6)	-2.74 ± 1.46 P = 0.001 (-5 ± 3)	P≠ = 0.045
**2 min oxygen consumption (mL·min)**	-10.2 ± 10.3 P = 0.006 (-140 ± 144)	-1.2 ± 8.1 P = 0.696 (-20 ± 96)	P≠ = 0.000	-16.8 ± 10.6 P = 0.000 (-250 ± 148)	-6.3 ± 11.6 P = 0.170 (-73 ± 136)	P≠ = 0.003	-15.1 ± 8.7 P = 0.000 (-231 ± 126)	-5.9 ± 11.9 P = 0.159 (-66 ± 145)	P≠ = 0.001
**2 min oxygen consumption (mL·kg·min^-1^)**	-9.8 ± 9.2 P = 0.004 (-2.1 ± 1.9)	-2.3 ± 8.3 P = 0.453 (-0.5 ± 1.6)	P≠ = 0.000	-16.9 ± 9.2 P = 0.000 (-3.7 ± 1.7)	-6.2 ± 12.2 P = 0.191 (-1.3 ± 2.4)	P≠ = 0.003	-16.0 ± 5.6 P = 0.000 (-3.5 ± 1.6)	-6.6 ± 12.5 P = 0.178 (-1.4 ± 2.5)	P≠ = 0.001
**4 min oxygen consumption (L·min)**	-5.4 ± 10.9 P = 0.068 (-85 ± 149)	1.9 ± 4.7 P = 0.289 (26 ± 68)	P≠ = 0.033	-10.7 ± 7.9 P = 0.002 (-162 ± 141)	-1.3 ± 3.9 P = 0.836 (-14 ± 51)	P≠ = 0.009	-6.8 ± 9.2 P = 0.021 (-116 ± 153)	-4.6 ± 9.2 P = 0.346 (57 ± 121)	P≠ = 0.412
**4 min oxygen consumption (mL·kg·min^-1^)**	-5.3 ± 9.3 P = 0.036 (-1.4 ± 1.9)	0.64 ± 5.4 P = 0.746 (0.2 ± 1.1)	P≠ = 0.071	-11.2 ± 6.7 P = 0.000 (-2.6 ± 1.6)	-1.22 ± 4.5 P = 0.471 (-0.2 ± 1.0)	P≠ = 0.003	-7.8 ± 8.7 P = 0.056 (-1.9 ± 1.9)	-5.4 ± 10.1 P = 0.173 (-1.3 ± 2.2)	P≠ = 0.414
**Resting heart rate (bpm)**	-2.5 ± 7.3 P = 0.261 (-2 ± 4)	-2.1 ± 9.2 P = 0.534 (-2 ± 6)	P≠ = 0.923	-3.0 ± 6.4 P = 0.149 (-2 ± 4)	-6.2 ± 7.7 P = 0.057 (-5 ± 5)	P≠ = 0.407	-2.2 ± 7.5 P = 0.335 (-2 ± 5)	-1.7 ± 11.1 P = 0.671 (-2 ± 7)	P≠ = 0.918
**Systolic BP (mm Hg)**	-1.0 ± 4.9 P = 0.508 (-1.0 ± 5.7)	-1.0 ± 2.4 P = 0.266 (-1.0 ± 2.4)	P≠ = 0.984	-2.0 ± 6.3 P = 0.293 (-2.3 ± 7.9)	-0.1 ± 3.9 P = 0.938 (0.0 ± 3.8)	P≠ = 0.459	-2.0 ± 6.6 P = 0.309 (-2.4 ± 8.0)	-0.3 ± 5.7 P = 0.888 (0.0 ± 6.1)	P≠ = 0.553
**Diastolic BP (mm Hg)**	-0.5 ± 5.9 P = 0.793 (-0.3 ± 4.4)	0.4 ± 4.5 P = 0.767 (0.1 ± 3.5)	P≠ = 0.704	-2.0 ± 6.4 P = 0.300 (-0.3 ± 4.4)	-0.7 ± 7.7 P = 0.809 (0.1 ± 3.5)	P≠ = 0.682	-2.2 ± 6.6 P = 0.268 (-1.8 ± 4.7)	-2.8 ± 5.8 P = 0.206 (-1.8 ± 4.1)	P≠ = 0.829

P value for difference in change within groups between 2 time points

P≠ value for difference in change between groups at each time point

**Table 3:** Effects of the exercise-training programme on physiological outcomes from interim time point – exercise group (n = 12); control group (n = 8)

	**% Δ Week 4 – 8**		**% Δ Week 8 – 12**	
**Variable**	**Exercise (mean ± SD)**	**Control (mean ± SD)**	**Exercise (mean ± SD)**	**Control (mean ± SD)**

**Peak oxygen consumption (mL·min)**	0.6 ± 5.0 P = 0.627 (15 ± 101)	-2.1 ± 8.5 P = 0.377 (-38 ± 154)	-1.3 ± 6.4 P = 0.377 (-33 ± 159)	-1.6 ± 7.9 P = 0.389 (-46 ± 166)
**Peak oxygen consumption (mL·kg·min^-1^)**	0.6 ± 5.8 P = 0.693 (0.1 ± 2.0)	1.0 ± 7.3 P = 0.015 (-0.3 ± 2.0)	-2.0 ± 9.6 P = 0.424 (-0.4 ± 2.8)	-2.9 ± 9.6 P = 0.685 (-1.1 ± 2.9)
**Time to exhaustion (min)**	4.0 ± 9.2 P = 0.190 (0.4 ± 1.0)	-0.5 ± 13.9 P = 0.826 (-0.2 ± 1.2)	-0.7 ± 12.8 P = 0.953 (-1.3 ± 1.8)	6.6 ± 22.9 P = 0.559 (0.3 ± 1.7)
**Peak heart rate (bpm)**	-1.28 ± 2.5 P = 0.096 (-2 ± 4)	-0.7 ± 2.5 P = 0.439 (-1 ± 5)	1.1 ± 1.7 P = 0.051 (2 ± 3)	-0.2 ± 1.9 P = 0.686 (-1 ± 4)
**2 min oxygen consumption (mL·min)**	-7.1 ± 8.3 P = 0.019 (-110 ± 146)	-5.0 ± 11.1 P = 0.217 (-53 ± 129)	2.4 ± 6.3 P = 0.275 (19 ± 73)	0.6 ± 7.9 P = 0.948 (7 ± 87)
**2 min oxygen consumption (mL·kg·min^-1^)**	-7.8 ± 8.6 P = 0.113 (-1.6 ± 1.8)	-4.0 ± 10.1 P = 0.286 (-0.8 ± 1.8)	1.9 ± 6.8 P = 0.363 (0.2 ± 1.2)	-0.2 ± 7.9 P = 0.902 (-0.1 ± 1.4)
**4 min oxygen consumption (L·min)**	-4.6 ± 7.0 P = 0.038 (-77 ± 136)	-2.9 ± 6.5 P = 0.398 (-39 ± 84)	3.6 ± 5.8 P = 0.049 (47 ± 78)	-3.1 ± 11.3 P = 0.441 (-43 ± 146)
**4 min oxygen consumption (mL·kg·min^-1^)**	-5.2 ± 6.9 P = 0.023 (-1.2 ± 1.7)	-1.7 ± 5.7 P = 0.381 (-0.4 ± 1.2)	3.4 ± 5.6 P = 0.009 (0.6 ± 1.0)	-4.0 ± 11.7 P = 0.339 (-1.0 ± 2.5)
**Resting heart rate (bpm)**	-0.1 ± 9.7 P = 0.846 (0 ± 6)	-3.7 ± 8.6 P = 0.254 (-3 ± 6)	1.1 ± 7.6 P = 0.700 (0 ± 5)	4.8 ± 9.0 P = 0.164 (3 ± 5)
**Systolic BP (mm Hg)**	-1.0 ± 5.3 P = 0.537 (-1.3 ± 7.0)	-0.9 ± 2.7 P = 0.368 (1.0 ± 2.9)	0.1 ± 4.5 P = 0.989 (-0.1 ± 5.1)	-0.2 ± 4.8 P = 0.915 (0.0 ± 5.1)
**Diastolic BP (mm Hg)**	-1.3 ± 6.3 P = 0.462 (-1.3 ± 5.3)	-1.2 ± 4.0 P = 0.455 (-0.7 ± 4.1)	-0.1 ± 5.6 P = 0.905 (-0.2 ± 4.2)	-1.6 ± 9.5 P = 0.539 (-1.2 ± 4.6)

P value for difference in change within groups between 2 time points

There was a significant time (F = 12.099; P = 0.000), and treatment (F = 5.456; P = 0.031) effect in % change for absolute 2minVO_2_, but no significant interaction effect (F = 2.385; P = 0.079). Post hoc analysis between groups revealed that significant differences occurred at weeks 4 (P = 0.000), 8 (P = 0.003) and 12 (P = 0.001) respectively. While post hoc analysis within groups showed significant reductions in the exercise group between week 0 and week 4 (P = 0.006), week 4 and week 8 (P = 0.019), week 0 and week 8 (P = 0.000), and week 0 and week 12 (P = 0.000) in the exercise group, but no significant changes within the control group at any time point.

There were significant time (F = 4.004; P = 0.012) and treatment effects (F = 4.803; P = 0.042), but no significant interaction effect (F = 2.705; P = 0.054) in % change for absolute 4minVO_2_. Post hoc analysis between groups revealed that significant differences occurred at weeks 4 (P = 0.033) and 8 (P = 0.009), but not at week 12. Significant reductions occurred in the exercise group between week 4 and week 8 (P = 0.038), week 0 and week 8 (P = 0.002), week 8 and week 12 (P = 0.049) and week 0 and week 12 (P = 0.021), but not between week 0 and week 4. No significant changes occurred at any time point in the control group.

### Changes in body composition and blood pressure

No significant time, treatment or interaction effects were observed for BMI (time F = 0.894; P = 0.364; treatment F = 0.468; P = 0.468; interaction F = 0.034; P = 0.857), weight (time F = 0.967; P = 0.389; treatment F = 0.501; P = 0.607; interaction F = 0.211; P = 0.652), systolic blood pressure (time F = 0.314; P = 0.746; treatment F = 1.657; P = 0.214; interaction F = 0.469; P = 0.641) or diastolic blood pressure (time F = 1.483; P = 0.229; treatment F = 0.293; P = 0.595; interaction F = 0.151; P = 0.929) over the 12 week intervention period.

### Changes in blood parameters

Table 4 and 5 present blood parameter outcomes. No significant time, treatment or interaction effects were observed for total cholesterol (time F = 0.145; P = 0.932; treatment F = 0.049; P = 0.827; interaction F = 0.769; P = 0.516), glucose (time F = 0.209; P = 0.890; F = 0.049; P = 0.827; F = 0.615; P = 0.608), IL-6 (time F = 0.877; P = 0.429; F = 2.482; P = 0.133; F = 1.326; P = 0.278) or TNF-α (time F = 0.057; P = 0.982; treatment F = 0.002; P = 0.961; interaction F = 1.180; P = 0.326) over the 12 week intervention period. However while there was no significant time or treatment effect for CRP in exercise and control groups (time F = 1.703; P = 0.201; treatment F = 0.189; P = 0.669), there was a significant interaction effect (F = 3.309; P = 0.027). Post-hoc analysis revealed that there were no significant differences between exercise and control groups at any of the time points tested. However there were significant reductions in CRP within the exercise group between week 1 and week 4 (P = 0.013), week 4 and week 8 (P = 0.000), and between week 1 and week 8 (P = 0.010), while there was no significant change at any time point in the control group. There was a trend for a decrease in TNF-α from baseline within the exercise group compared to the control group.

**Table 4:** Effects of the exercise-training programme on blood parameters from baseline – exercise group (n = 12); control group (n = 8)

	Δ **Week 1 – 4**			Δ **Week 1 – 8**			Δ **Week 1 – 12**		
**Variable**	**Exercise (mean ± SD)**	**Control (mean ± SD)**	**Difference between groups**	**Exercise (mean ± SD)**	**Control (mean ± SD)**	**Difference between groups**	**Exercise (mean ± SD)**	**Control (mean ± SD)**	**Difference between groups**

**Total Cholesterol (mmol/L)**	0.0 ± 0.6 P = 0.827	0.0 ± 0.5 P = 0.880	P≠ = 0.688	-0.2 ± 0.4 P = 0.136	0.1 ± 0.3 P = 0.590	P≠ = 0.771	0.0 ± 0.4 P = 0.967	0.0 ± 0.5 P = 0.944	P≠ = 0.692
**Total Glucose (mmol/L)**	0.1 ± 1.0 P = 0.416	-0.1 ± 0.4 P = 0.943	P≠ = 0.934	0.0 ± 0.8 P = 0.912	0.1 ± 0.6 P = 0.450	P≠ = 0.511	-0.1 ± 0.9 P = 0.936	-0.2 ± 0.6 P = 0.844	P≠ = 0.760
**IL-6 (pg/L)**	-0.3 ± 1.0 P = 0.269	0.7 ± 0.8 P = 0.038	P≠ = 0.939	-0.7 ± 2.0 P = 0.231	0.3 ± 1.2 P = 0.553	P≠ = 0.974	0.2 ± 1.2 P = 0.660	-0.1 ± 0.7 P = 0.840	P≠ = 0.324
**TNF-α (pg/L)**	-0.9 ± 1.3 P = 0.032	0.8 ± 2.4 P = 0.363	P≠ = 0.448	-0.9 ± 1.7 P = 0.102	0.3 ± 1.6 P = 0.663	P≠ = 0.297	-0.9 ± 1.6 P = 0.086	0.3 ± 1.3 P = 0.567	P≠ = 0.268
CRP (mg/L)*	-0.4 ± 0.6 P = 0.013	-0.3 ± 0.9 P = 0.526	P≠ = 0.585	-0.9 ± 0.8 P = 0.010	-0.4 ± 1.3 P = 0.127	P≠ = 0.224	-1.2 ± 1.5 P = 0.823	0.1 ± 0.7 P = 0.836	P≠ = 0.199

P value for difference in change within groups between 2 time points

P≠ value for difference in change between groups at each time point

CRP (mg/L)* P value based on logged data transformation

**Table 5:** Effects of the exercise-training programme on blood parameters from interim time point – exercise group (n = 12); control group (n = 8)

	Δ **Week 4 – 8**		Δ **Week 8 – 12**	
**Variable**	**Exercise (mean ± SD)**	**Control (mean ± SD)**	**Exercise (mean ± SD)**	**Control (mean ± SD)**

**Total Cholesterol (mmol/L)**	-0.2 ± 0.6 P = 0.365	0.1 ± 0.3 P = 0.464	-0.2 ± 0.5 P = 0.170	-0.1 ± 0.3 P = 0.667
**Total Glucose (mmol/L)**	-0.1 ± 0.2 P = 0.195	0.1 ± 0.4 P = 0.480	0.0 ± 0.2 P = 0.955	-0.1 ± 0.4 P = 0.388
**IL-6 (pg/L)**	-0.4 ± 1.2 P = 0.306	-0.4 ± 1.3 P = 0.361	0.9 ± 1.5 P = 0.077	-0.3 ± 0.1 P = 0.338
**TNF-α (pg/L)**	0.1 ± 1.5 P = 0.894	-0.6 ± 1.5 P = 0.319	0.0 ± 1.7 P = 0.945	0.0 ± 1.2 P = 0.977
CRP (mg/L)*	-1.0 ± 0.4 P = 0.000	0.0 ± 0.5 P = 0.266	-0.5 ± 0.7 P = 0.101	0.6 ± 1.74 P = 0.284

P value for difference in change within groups between 2 time points

CRP (mg/L)* P value based on logged data transformation

## Discussion

The data from the study confirmed that a moderate intensity aerobic exercise-training programme performed 4 times a week could be successfully implemented within the workplace during working hours. Furthermore, it was demonstrated that it was effective at reducing risk factors associated with cardiovascular disease, and at improving physiological capacity within previously sedentary individuals. Specifically, significant improvements were found in peak oxygen consumption (VO_2 peak_), economy of absolute oxygen utilization at both 2 minutes (2minVO_2_) and 4 minutes (4minVO_2_), and C-reactive protein (CRP) concentration. These results confirm previous reports showing that improved cardiovascular fitness, or physical activity level reduces cardiovascular risk, with a particular association with lower CRP levels [9,23,24]. This is the first report combining objective physiological outcome measures with objective monitoring of the training programme to demonstrate the type of exercise that can be effectively carried out during working hours, while still providing health related benefits.

At the end of the 8-week intervention period absolute VO_2 peak _increased significantly by 5 % in the exercise group, while it decreased significantly by 6 % in the control group. There was no significant change in peak heart rate in the exercise group, but there was a significant reduction in peak heart rate in the control group, suggesting that a decline in effort contributed to the observed fall in VO_2 peak_. Absolute 2minVO_2 _and 4minVO_2 _decreased significantly by 17 % and 11 % respectively in the exercise group, while there was no significant change in the control group. Furthermore, as the exercise group averaged the completion of 81 % and 84 % of the prescribed exercise sessions between week 1 and week 4, and week 4 and week 8 respectively, it can be concluded that the progressive aerobic exercise training programme was not only effective at improving the physical fitness of a sedentary group of adults, but was also successful at increasing physical activity levels.

However although cardiovascular fitness and physical activity are positively related, research indicates that it is the former that is more closely linked to cardiovascular disease risk factors and disease, than actual physical activity level [25,26]. As a consequence it has been shown that it is only those individuals who increase their VO_2 max_, rather than their actual physical activity level that reduce their relative risk of cardiovascular disease risk factors [27]. This has been attributed to a reduction in large artery stiffness, which may be mediated by concomitant changes in high-density lipoprotein (HDL) cholesterol and body weight [28].

This holds relevance for the present study: after 8 weeks when the exercise group were not provided with any further progression or instruction to the exercise training programme VO_2 peak _decreased by 2 %. In view of the 70 % completion of the 16 sessions, and the significant improvement in absolute 4minVO_2 _(-7 %), it appears probable that the intensity of the exercise performed within this time period was too low to challenge VO_2 peak_. This is supported by evidence that indicates that VO_2 max _has a modest association with physical activity, but a much stronger association with the mean intensity of the exercise [29]. In view of this, and the cardio protective benefit of an increase in VO_2 max _future research should evaluate the implication of a higher intensity workplace exercise training programme on the modification of cardiovascular risk profile, while assessing whether it remains successful at ensuring exercise adherence.

It appears that supervision and progression of the exercise programme may influence adherence [30,31]. In the present study, at 8 weeks when no further progression or supervision to the exercise training programme was provided a reduction in the adherence of the training sessions occurred; 81 % and 84 % were completed in week 1 to week 4 and week 4 to week 8, while only 70 % were completed in week 8 to week 12. This could further highlight the need for employers to ensure the provision of additional support and progression to the original training programme for optimal participation of employees, and success of the programme.

The exercise group demonstrated a significant decrease in CRP of -0.4 ± 0.6 mg/L between week 1 and week 4, and -1.0 ± 0.4 mg/L between week 4 and week 8. However while this is in accordance with previous research [24,32], it should be noted that due to a mean baseline value indicating high risk for CVD (> 3.0 mg/L), that the reduction would still result in a mean value indicating average risk of CVD (2.2 mg/L) [33]. The mechanism behind such action remains unclear. It has been postulated that a reduction in CRP is attained via the positive benefit of exercise on BMI via modulation of the percentage of visceral fat and insulin receptor sensitivity [24]. However, within the present study there was no such positive effect on body composition, or fasting glucose. Another potential explanation is that among unfit individuals there is a greater generation of reactive oxygen species via normal metabolic processes, and unaccustomed muscle stretching. This leads to subliminal injury of the myocytes, that causes both cell and tissue oxidative damage, leading to an inflammatory response [34]. Evidence confirms that chronic exercise induces a mechanical resistance of the myocytes to stretching, and elevates endogenous antioxidant enzyme activity, which prevents excessive local inflammatory response [35]. As there were significant gains in aerobic capacity within the exercise group it is plausible that this explanation provides a mechanism of action for the observed results.

No significant change was observed in IL-6 at any time point during the study. However there was a significant reduction in TNF-α between week 1 and week 4 in the exercise group. As TNF-α directly impairs glucose uptake and metabolism via a direct effect on insulin signal transduction, a reduction holds positive benefit for prevention of CVD [10]. Thus despite the lack of a significant change in fasting glucose, there is still suggestive evidence that the training programme may accrue positive benefit for this specific risk factor.

Although the present study was successful at improving maximal and submaximal aerobic exercise capacity, it had no significant effect on fasting glucose or cholesterol, blood pressure or BMI. It is likely that the small sample size is responsible for such null findings. However it is also unsurprising for a number of reasons.

Firstly, although physical activity and exercise improves insulin sensitivity through a direct effect on the muscle (enhancement of insulin receptor autophsophorylation [36], increase in GLUT-4 content [37] and glucose transport-phosphorylation [38], and a reduction in visceral obesity [39], neither the exercise nor the control group exhibited impaired glucose tolerance (exercise = 5.04 ± 0.50; control = 5.11 ± 0.52 mmol/L) at baseline that would have required intervention modification. The same can be said for blood pressure, with all participants classified as normotensive (exercise = 118 ± 12/73 ± 10; control = 106 ± 10/69 ± 9) at baseline. Nevertheless, in view of the beneficial effect that exercise has on glucose tolerance, and evidence that those with low levels of physical fitness are shown to be at a relative risk of 1.52 for developing hypertension, when compared to highly fit individuals [6], the use of exercise in aiding glycemic control, and the maintenance of healthy blood pressure should still be encouraged.

Secondly, regarding BMI, it should be considered that the aim of the training programme was not to directly target weight loss for a reduction of cardiovascular risk, but instead to improve physiological capacity, and biomarkers of cardiovascular profile. In accordance with this, and in the absence of dietary modification, it would have been unlikely that the 4 × 30 minute sessions per week would have provided the necessary negative energy balance stimulus of 500 – 1000 kcal·d^-1 ^to achieve gradual weight loss (ACSM, 2006). Given that a BMI ≥ 30 kg·m^-2^classifies obesity, concomitantly increasing the risk of hypertension, poor total cholesterol/HDL cholesterol ratio, coronary disease and mortality rate [40], there is a need for future work place health promotion programmes to evaluate whether an aerobic exercise training programme specifically targeting weight loss and management as its primary outcome can be successfully implemented within the workforce.

A limitation of the present study was the failure to examine lipoprotein subfractions; small low-density lipoproteins (LDLs), high-density lipoproteins (HDLs), high-density lipoprotein subfractions (HDL_3 _and HDL_2_), very low-density lipoproteins (VLDLs), and respective particle size, that better reflect CVD risk than absolute measures of cholesterol concentrations [41]. In a recent study, Halverstadt et al (2007) concluded that an aerobic exercise training program consisting of 20 minutes, 3 days a week, progressively building up to a duration of 40 minutes and an intensity of 70 % VO_2 max _for a period of 24 weeks, plus a weekend walk was successful at improving lipid subfraction profile and cardiovascular risk independent of diet and change in body fat. This is supported by several other studies, which also indicate an improved plasma lipoprotein profile with exercise training, exclusive of weight loss [5,42].

## Conclusion

Our pilot study provides objective and randomised controlled trial data demonstrating that regular supervised exercise increases physical activity for healthy individuals, and improves exercise capacity, with a concomitant cardioprotective benefit. As this can be achieved without disrupting the working day, this exercise programme provides a means of improving health at work. As the study was conducted within an NHS department, it may be of particular relevance to the NHS, as the single largest employer in Europe.

## Competing interests

The author(s) declare that they have no competing interests.

## Authors' contributions

JAH conceived the study design, carried out the testing, performed statistical testing and drafted the manuscript. MM carried out the immunoassays. GPW participated in the coordination of the study and drafting of the manuscript. KvS helped to draft the manuscript. TSL conceived of the study, and participated in its design and coordination, and helped to draft the manuscript. All authors read and approved the final manuscript.
